# DPP-IV Inhibitory Potentials of Flavonol Glycosides Isolated from the Seeds of *Lens culinaris*: In Vitro and *Molecular Docking* Analyses

**DOI:** 10.3390/molecules23081998

**Published:** 2018-08-10

**Authors:** Bo-Ram Kim, Hyo Young Kim, Inhee Choi, Jin-Baek Kim, Chang Hyun Jin, Ah-Reum Han

**Affiliations:** 1 Advanced Radiation Technology Institute, Korea Atomic Energy Research Institute, Jeongeup-si, Jeollabuk-do 56212, Korea; boram1606@kaeri.re.kr (B.-R.K.); khy5012@kaeri.re.kr (H.-Y.K.); jbkim74@kaeri.re.kr (J.-B.K.); chjin@kaeri.re.kr (C.H.J.); 2 Institut Pasteur Korea, Seongnam-si, Gyeonggi-do 13488, Korea; inhee.choi@ip-korea.org

**Keywords:** *Lens culinaris*, flavonol glycoside, dipeptidyl peptidase IV, diabetes, molecular docking analysis

## Abstract

Dipeptidyl peptidase IV (DPP-IV), a new target for the treatment of type 2 diabetes mellitus, degrades incretins such as glucagon-like peptide 1 (GLP-1) and glucose-dependent insulinotropic polypeptide. DPP-IV inhibitors shorten the inactivation of GLP-1, permitting the incretin to stimulate insulin release, thereby combating hyperglycemia. In our ongoing search for new DPP-IV inhibitors from medicinal plants and foods, three flavonol glycosides (**1**–**3**) were isolated from the seeds of *Lens culinaris* Medikus (Fabaceae) and tested for their DPP-IV–inhibitory activity. We demonstrated for the first time, that compounds **1**–**3** inhibited DPP-IV activity in a concentration-dependent manner in our in vitro bioassay system. In addition, molecular docking experiments of compounds **1**–**3** within the binding pocket of DPP-IV were conducted. All investigated compounds readily fit within the active sites of DPP-IV, in low-energy conformations characterized by the flavone core structure having optimal electrostatic attractive interactions with the catalytic triad residues of DPP-IV.

## 1. Introduction

Type 2 diabetes mellitus is an acquired syndrome characterized by several defects in the regulation of glucose homeostasis, including elevated blood glucoside levels, increased hepatic glucose production, deficient insulin secretion, insulin resistance, and/or pancreas β-cell dysfunction [1,2]. As reported by the World Health Organization [3], type 2 diabetes mellitus comprises approximately 90% of all cases of diabetes, and an estimated 15 million people globally have type 2 diabetes mellitus, a figure that could double by 2025. Recently, incretin-based therapy has emerged for the treatment of type 2 diabetes mellitus. The incretin system comprises glucagon-like peptide-1 (GLP-1) and glucose-dependent insulinotropic polypeptide (GIP), which stimulate the release of insulin from pancreatic β-cells, in response to high blood glucose concentrations [2]. Dipeptidyl peptidase IV (DPP-IV), is a serine protease ectoenzyme that is present in the gastrointestinal tract, kidneys, and endothelial layer of blood vessels, and it contributes to the regulation of various physiological processes, such as blood glucose homeostasis by cleaving peptide hormones, chemokines, and neuropeptides [4]. In the incretin system, DPP-IV is responsible for the rapid degradation and inactivation of GLP-1 and GIP [5]. Consequently, DPP-IV inhibition improves glucose tolerance in patients with diabetes by enhancing the insulin-producing effects of GLP-1 [6]. Furthermore, GLP-1 operates in a glucose-dependent manner, reducing the risk of hypoglycemia and stimulating the proliferation of insulin-producing β-cells, providing additional benefits for patients with diabetes [7]. Consequently, intense research efforts have been devoted to identifying novel DPP-IV inhibitors, for the treatment of type 2 diabetes. Given the current state of DPP-IV inhibitors, most synthetic compounds are used in current drug therapies and promising drug candidates, which have demonstrated significant modulatory effects on DPP-VI enzymes continue to be developed [8].

*Lens culinaris* Medikus is a pulse crop of the family Fabaceae, and its edible seed is known as lentil. Lentil has long been cultivated and used as foodstuff in Europe, the Middle East, Africa, and Asia [9]. Lentil was reported to have diverse biological activities such as antioxidant [9,10], α-glucosidase-inhibitory [10], anti-inflammatory [11], and anticancer effects [12]. In a previous phytochemical study, phenolics, flavonoids, tannins, saponins, and fatty acids were isolated from *L. culinaris* [9,13,14,15].

In our ongoing search for DPP-IV inhibitors in natural products, the *n*-butanol-soluble fraction of the seeds of *L. culinaris* was investigated, and three known flavonol glycosides were isolated. All isolates were tested for their DPP-IV-inhibiting activities, using an in vitro bioassay with human recombinant DPP-IV. In this study, we described the isolation and structure identification of these compounds and suggested potent anti-diabetic candidates.

## 2. Results and Discussion

### 2.1. Isolation of Compounds ***1***–***3*** from the Seeds of L. culinaris

**Figure 1:**
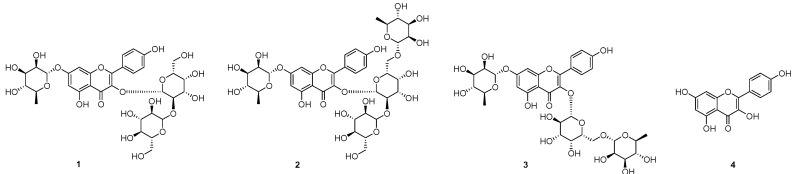
Chemical structures of compounds **1**–**3** isolated from the seeds of *Lens culinaris* and kaempferol (**4**).

The phytochemical investigation of the seeds of *L. culinaris* lead to the isolation of compounds **1**–**3**. Their structures were identified as kaempferol-3-*O*-β-gulcopyranosyl-(1→2)-β-galactopyranosyl-7-*O*-α-rhamnopyranoside (**1**) [16], kaempferol-3-*O*-β-gulcopyranosyl-(1→2)-[α-rhamnopyranosyl(1→6)]-β-galactopyranosyl-7-*O*-α-rhamnopyranoside (**2**) [17], and robinin (kaempferol-3-*O*-α-rhamnosyl(1→6)-*O*-β-galactopyranoside-7-*O*-α-rhamnopyranoside; **3**) [17], via analysis of their spectroscopic data, as well as comparisons, of their data with published values, as shown in Table S1 and Figure 1. 

### 2.2. DPP-IV Inhibitory Activity of ***1***–***4***

**Figure 2:**
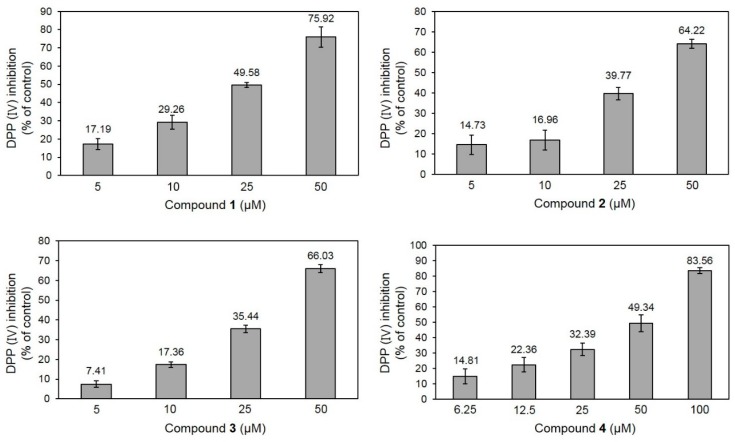
Effects of compounds **1**–**4** on dipeptidyl peptidase IV (DPP-IV) activity. Values are presented as the mean ± SD of three independent experiments.

An in vitro bioassay for DPP-IV inhibition using human recombinant DDP-IV was performed in the absence or presence of compounds **1**–**3** (5, 10, 25, and 50 μM) and the commercial compound kaempferol (**4**), which comprised the aglycone backbone of these compounds (6.25, 12.5, 25, 50, and 100 μM). Compounds **1**–**4** inhibited DPP-IV in a concentration-dependent manner in this assay system, as shown in Figure 2. Their IC_50_ values, as determined using a linear standard curve and calculated based on the molecular mass of each compound, were 27.89 ± 1.29, 36.52 ± 0.78, 37.01 ± 1.40, and 51.9 ± 4.83 μM, respectively. The positive control sitagliptin, had an IC_50_ of 0.071 ± 0.005 μM. To the best of our knowledge, no prior studies examined the in vitro DPP-IV-inhibitory effects of **1**–**3**, although the protein-ligand complex for **3** was examined via molecular docking analysis, using Discovery Studio 3.0 and Meta-Pocket (http://projects.biotec.tu-dresden.de/metapocket/) [18]. Compound **4** was previously reported to have greater DPP-IV–inhibitory activity with an IC_50_ of 0.49 ± 0.02 μM using a DPP-IV Glo™ Protease Assay kit (Promega, Madison, WI, USA), as compared with the activity result using our assay kit [19]. Additionally, **4** also inhibited DPP-IV activity by binding to the active site of the enzyme, in a molecular docking analysis using AUTODOCK 4.2 (CCDC, UK; http://www.ccdc.cam.ac.uk/products/cds) [19].

In the reports on recent progress of DPP-IV inhibitors from natural product [20,21], lots of active chemical components of natural sources were found with potent DPP-IV inhibitory effects, which will have a potential of valuable leads to develop safe DPP-IV inhibitors. In the flavonoid group, which is the same category as the isolated compound structures in this study, eighteen flavonoids, isolated citrus, berry, grape, soybean, or cotton flower, were found to have remarkable DPP-IV inhibitory activities within the range of IC_50_ values of 0.12 to 96.8 μM [20]. Among them, isoquercitrin and naringin demonstrated strong in vivo bioactivities as a DPP-IV inhibitor [22,23]. Therefore, our results suggested the potential of **1**–**3** as naturally occurring agents for treating DPP-IV-mediated hyperglycemia and type 2 diabetes mellitus, although further studies are required to clarify their mechanisms of action using in vitro and in vivo models.

### 2.3. Molecular Docking Analysis of Compounds ***1***–***4***

Based on the suggested mechanism of the hypoglycemic action of kaempferol, we examined possible binding interactions between kaempferol derivatives and DPP-IV.

For docking, we used the structure of a recombinant, soluble form of human DPP-IV that begins at residue Ser39; corresponding to the predominant form found in human plasma. The X-ray structure used for docking in this study is 1X70 (PDB ID) [24], which is complexed with sitagliptin. It is reported in the PDB file that residue 39, the starting residue of this X-ray structure, has been engineered to Thr from Ser. Each subunit consists of two domains, namely an α/β-hydrolase domain and an eight-bladed β-propeller domain [4]. Between these two domains, a large cavity with an estimated volume of approximately 5.968 Å^3^ is found.

DPP-IV has three binding pockets/active sites (S1, S2, and S3). S1 consists of Tyr547, Ser630, Tyr631, Val656, Trp659, Tyr662, Tyr666, Asn710, Val711, and His740, all of which are involved in strong hydrophobic interactions. Among these residues, Ser630, Asn710, and His740 form the catalytic triad. S2 is the cavity near Glu205, Glu206, and Tyr662. S3 consists of Ser209, Arg358, and Phe357 [19].

Prior to docking each compound, sitagliptin bound in the DPP-IV X-ray structure was subjected to reproducibility testing. It was removed from the structure and re-docked within the defined grid. The root-mean-square deviation between docked sitagliptin and that of the X-ray structure was 0.876 Å. Sitagliptin bound to the active site of DPP-IV with great specificity, covering all three binding pockets, specifically situating its trifluorophenyl moiety within S1, forming four hydrogen bonds with residues of S2, and burying its trifluoro group within the extremely tight pocket of S3. All critical interactions with key residues (Phe357, Glu205, Glu206, Tyr666) and HOH 1986, were identical with an additional hydrogen bond between Asp663 and HOH 1957 (data not shown).

**Figure 3:**
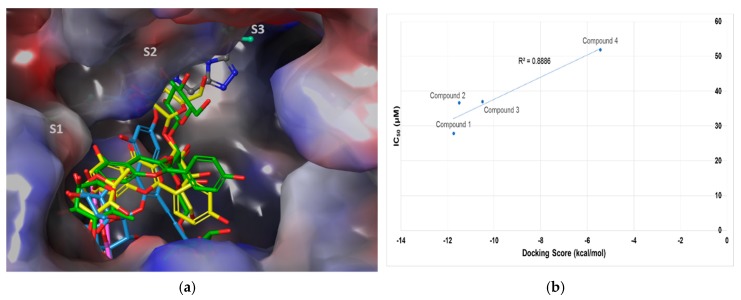
(**a**) The binding poses of all docked compounds in the binding pockets/active sites of human dipeptidyl peptidase IV. The binding pockets/active sites are labeled as S1, S2, and S3 (light grey). They are shown on the electrostatic surface. Sitagliptin is shown partially bound in the S3 pocket (grey atom-colored stick), and compounds 1 (yellow), 2 (green), 3 (light blue), and 4 (pink) are all docked into the wide S1 and S2 pockets. (**b**) Linear correlation between the docking scores and IC_50_ values, with R^2^ = 0.8886 for compounds **1**–**4**.

All compounds subjected to docking fit well within the wide cavities of S1 and S2. The docking score and interaction results, are listed in Table 1. Sitagliptin was located mainly in S2 and partially in S3, whereas the natural products were positioned in both S1 and S2 (Figure 3a). The flavone core structure of all compounds was mainly located in S1, and the glycosides of **1**–**3** interacted with residues in S2. When the docking simulation data were compared with the results of the experimentally validated in vitro bioassay for human recombinant DPP-IV inhibition, the correlation plot of docking scores and IC_50_ values of docked compounds, indicated a good correlation with an R^2^ of 0.889, as shown in Figure 3b.

**Figure 4:**
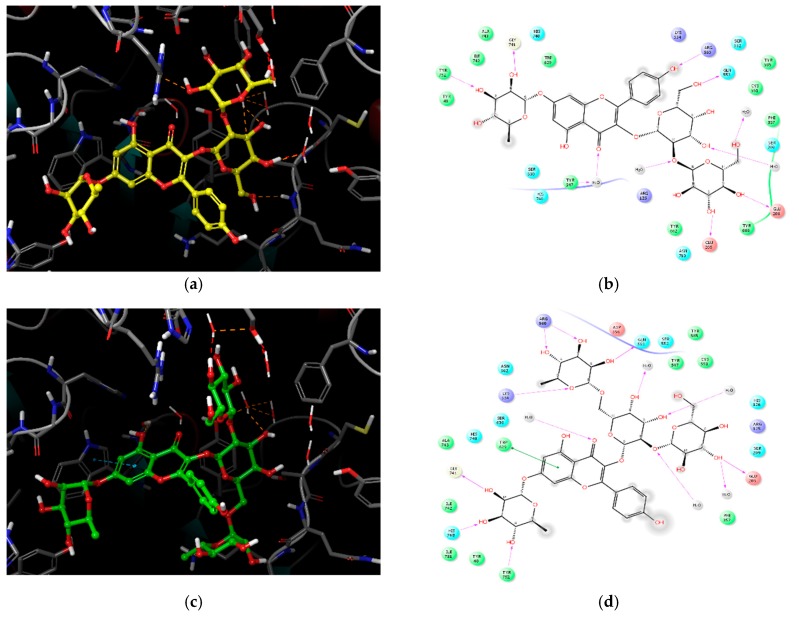

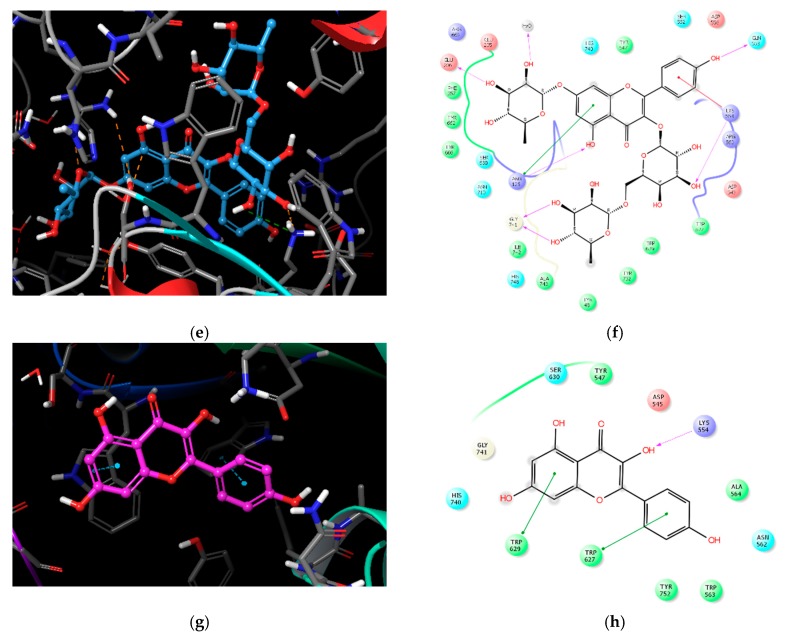
Docking poses of compounds **1**–**4** in the dipeptidyl peptidase IV active site and their ligand interaction diagrams (LIDs). (**a**) Docking pose of compound **1**, (**b**) LID of compound **1**, (**c**) docking pose of compound **2**, (**d**) LID of compound **2**, (**e**) docking pose of compound **3**, (**f**) LID of compound **3**, (**g**) docking pose of compound **4**, and (**h**) LID of compound **4**. All interacting residues and water molecules are shown in the atom-colored stick model. All compounds are represented in the ball-and-stick model. Orange dotted lines indicate hydrogen bonds, and blue dotted lines denote π–π interactions. Only polar hydrogens in compounds are shown for clarity. In the LIDs, solid magenta arrows indicate hydrogen bonds with backbones, dashed magenta arrows indicate hydrogen bonds with side chains, green lines indicate π–π stacking, and red lines represent π–cation stacking. Residues are colored in circles according to their properties: (1) orange for negatively charged compounds, (2) purple for positively charged compounds, (3) green for hydrophobic compounds, (4) blue for polar compounds, (5) ivory for glycines, and (6) grey for water molecules.

Compound **1**, which had the best inhibitory activity, had the lowest docking score of −11.737 kcal/mol, as shown in Table 1 and Figure 4a,b. The flavone core structure interacted closely with the key residues of site S1, namely Ser630 and His740. Tyr547 formed a water-mediated hydrogen bond with the C-ring carbonyl group of the flavone core. The glucopyranoside and galactopyranoside moieties of the 3-*O*-β-glucopyranosyl-(1→2)-β-galactopyranosyl group in **1** were positioned in the S2 pocket, in which hydroxyls exhibited favorable H-bond interactions with Glu205, Glu206, Gln553, and water molecules. The rhamnopyranoside moiety positioned at C-7 of **1** interacted well with the residues in the S1 pocket.

Compound **2**, which possesses four sugar moieties, had the second strongest inhibitory activity. It had a docking score of −11.499 kcal/mol, as shown in Table 1 and Figure 4c,d. The flavone core oriented itself by interacting with the catalytic triad residues in the S1 pocket. The three sugar moieties attached at the 3 position of the flavone core docked well into S1 and S2. Several hydroxyl groups formed hydrogen bonds with hydrophilic residues, such as Glu205, Gln553, Arg560, and water molecules. The glucopyranoside extended toward the S3 pocket near Ser209 and Phe357. These stronger hydrophilic interactions could have influenced the B-ring of the flavone core to face toward the empty space, causing slightly less interaction between the core structure and DPP-IV residues, compared with the findings for compound **1**.

Compound **3**, which is structurally similar to compound **1**, docked into the S1 and S2 pockets, with a docking score of −10.494 kcal/mol (Table 1 and Figure 4e,f). The difference in the sugar moieties rhamnopyranoside and galactopyranoside, explained the difference in interactions, compared with the findings for compound **1**. The orientation of the flavone core slightly differed from that of **1** and **2**, due to the hydrophilic interactions with residues in the S1 pocket of the 3 position in rhamnopyranoside and galactopyranoside. Conversely, the sugar moieties in the 3 position of compounds **1** and **2**, were positioned mainly within the S2 pocket, stretching out toward S3.

Finally, compound **4**, a small aglycone lacking sugar groups, stably docked in the S1 pocket with the highest docking score of −5.439 kcal/mol (Table 1 and Figure 4g,h). The 3-hydroxyl group formed a hydrogen bond with Lys554. The B-ring in the flavone core had a strong π–π interaction with Trp627, and hydrophobic interactions with Trp563 and Tyr752. This caused the flavone core to tilt perpendicularly, relative to the other compounds forming π–π interactions with Trp629. This slight difference in the orientation of the flavone core structure and less coverage of the active sites, could explain the lower activity of this compound against human DPP-IV.

## 3. Materials and Methods 

### 3.1. General Procedures

^1^D-NMR was performed using a JNM-ECA 500MHz NMR instrument (JEOL Ltd., Tokyo, Japan). LC/ESI-MS was conducted using an Agilent 1200 series system and an Agilent 6120 quadrupole MS system (Agilent Technologies Co., Santa Clara, CA, USA). Thin-layer chromatography was performed using Kieselgel 60 F254 plates (Merck, Darmstadt, Germany), with visualization performed under UV light (254 and 365 nm) and 10% (*v*/*v*) sulfuric acid spray followed by heating (200 °C, 2 min). YMC Gel ODS-A (12 nm, S-150 μM; YMC Co., Kyoto, Japan) and Sephadex LH-20 columns (Pharmacia Co., Uppsala, Sweden) were used for column chromatography (CC). Kaempferol (≥97.0%, HPLC) was purchased from Sigma-Aldrich (St. Louis, MO, USA). All other chemicals and solvents used in this study were of analytical grade.

### 3.2. Plant Materials

The seeds of *L. culinaris* (Austgrains Pty Ltd., Moree, New South Wales, Australia) were purchased from a local market in Jeongeup-si, Jeollabuk-do, Korea. The voucher specimens (No. Con011) have been deposited at the Radiation Breeding Research Center, Advanced Radiation Technology Institute, Korea Atomic Energy Research Institute.

### 3.3. Extraction and Isolation

The dried seeds of *L. culinaris* (8 kg) were pulverized and extracted using 95% EtOH (3 × 15 L), overnight at room temperature. The solvent was evaporated *in vacuo* to afford a 95% EtOH extract (210 g), which was then suspended in distilled water (1 L) and partitioned with *n*-hexane (2 × 3 L), chloroform (2 × 3 L), ethyl acetate (1 L), and *n*-butanol (1 L), sequentially. The *n*-butanol-soluble fraction (2.3 g) was subjected to RP-C_18_ CC (MeOH-water, 1:1 to 1:0, *v*/*v*) to yield seven fractions (F01–F07). Fraction F02 (1.5 g) was subjected to RP-C_18_ CC (MeOH-water, 1:2, *v*/*v*) to give three sub-fractions (F0201–F0203). Sub-fraction F0202 (160.8 mg) was chromatographed on a Sephadex LH-20 column (100% MeOH), providing **3** (16.5 mg). Sub-fraction F0203 (25.9 mg) was chromatographed on a Sephadex LH-20 column (100% MeOH), furnishing **1** (15.0 mg) and **2** (1.0 mg).

### 3.4. DPP-IV–Inhibitory Activity Assay

DPP-IV activity was analyzed using a DPP-IV inhibitor screening assay kit (Cayman Chemical, Ann Arbor, MI, USA), which provided a fluorescence-based method for screening DPP-IV inhibitors. The assay used the fluorogenic substrate, Gly-Pro-Aminomethylcoumarine (AMC), to measure DPP-IV activity. Cleavage of the peptide bond by DPP released the free AMC group, resulting in fluorescence that could be analyzed using an excitation wavelength of 350–360 nm and an emission wavelength of 450–465 nm.

The tested compounds were initially dissolved in DMSO to produce 50 mM stock solution, and subsequently diluted to the required concentrations using DMSO, and then they were added to a 96-well plate in final volume of 10 μL and a final concentration of 50 μM. The assay procedure is described briefly according to the manufacturer’s protocols as follows: Diluted assay buffer (30 μL) and diluted enzyme solution (10 μL) were added to the 96-well plate containing 10 μL of solvent (blank) or solvent-dissolved test compounds. The reaction was initiated by adding 50 μL of a diluted substrate solution, and the plate was incubated for 30 min at 37 °C. After incubation, fluorescence with an excitation wavelength of 350 nm and an emission wavelength of 450 nm was monitored using a plate reader (TECAN, Männedorf, Switzerland). The percent inhibition was expressed as ([DPP-IV level of vehicle–treated control–DPP-IV level of test samples]/DPP-IV level of vehicle-treated control) × 100.

### 3.5. Molecular Docking

The selected X-ray crystal structure (PDB ID 1X70) of human DPP-IV complexed with the well-known drug sitagliptin, was prepared using the protein preparation wizard of the Maestro software package from the Schrödinger suite (release version 2016–3, New York, NY, USA) [25]. The A chain of this protein was prepared for docking in which water molecules within 5 Å of sitagliptin were included, and the hydrogens were added.

The ligands were prepared using the LigPrep module of Schrodinger-9.2. The 2D structures of selected compounds were drawn and then prepared in 3D for docking using the default values of the ligand preparation tool (release version 2016–3). The chiralities were retained as drawn. The structures and the inhibitory values of each compound, are shown in Table 1.

Protein-ligand simulations were performed using the extra precision algorithm of Glide [26]. The grid points were generated to encompass the S1 and S2 pockets of DPP-IV. The grid box was generated by controlling the grid size, such that it was sufficient to accommodate large natural products from the prepositioned sitagliptin as centroid. The following option for docking was selected: “Reward intramolecular hydrogen bonds”. The best poses of each compound were selected based on the docking score, as well as the interactions of critical residues, within ≤4.0 Å of the docked position.

## 4. Conclusions

In conclusion, we reported the inhibitory effects of flavonol glycosides, particularly compounds **1**–**3** isolated from the seeds of *L. culinaris*, against human recombinant DPP-IV for the first time. As referred to in the report, the S1 and S2 pockets are crucial for recognizing ligands as DDP-IV inhibitors [18]; compounds **1**–**3** and their aglycone core **4** docked into both S1 and S2, in which the flavone core structure was stably positioned in the S1 pocket, interacting with the catalytic triad residues. Glycosides in compounds **1**–**3** interacted with residues in the S2 pocket to accommodate the structurally large ligands of DPP-IV. Overall, hydrogen bonding including water-mediated binding was the primary binding mode of all tested compounds with DPP-IV. Therefore, these compounds can act as naturally occurring DPP-IV inhibitors given their ability to bind directly to the active sites of DPP-IV.

## Figures and Tables

**Table 1:** Dipeptidyl peptidase IV inhibition by compounds **1**–**4**, as well as their docking energy and interaction types.

Compound	IC_50_ (μM) ^1^	Number of OH Groups	Docking Score (kcal/mol)	Interacting Residues (≤4.0 Å) ^2^
**1**	27.89 ± 1.29	12	−11.737	H-bond: E205, E206, Q533, Y547 (water-mediated), R560, G741, Y752, HOH1605, HOH1617, HOH1927, HOH1957 VDW: W629, S630
**2**	36.52 ± 0.78	14	−11.499	H-bond: E205, Q553, K554, R560 (2), G741, H748, Y752, HOH1582, HOH1617, HOH1732, HOH1927, HOH1957 π–π: W629
**3**	37.01 ± 1.40	11	−10.494	H-bond: R125, E206, Q553, K554, G741(2), HOH1582 π–cation: K554 VDW: R125
**4**	51.69 ± 4.83	4	−5.439	H-bond: K554 π–π: W627, W629

^1^ Values are expressed as the mean ± SD of three independent experiments. ^2^ H-bond: Hydrogen bonding; VDW: van der Waals interaction.

## Supplementary Materials

The following are available online, Table S1: ^1^H-NMR data of compounds **1**–**3** (500 MHz, δ, ppm), Table S2: ^13^C-NMR data of compounds **1**–**3** (125 MHz, δ, ppm).
