# Supplementary material for: DPP-IV Inhibitory Potentials of Flavonol Glycosides Isolated from the Seeds of Lens culinaris: In Vitro and Molecular Docking Analyses
Source: Molecules. 2018 Aug 10;23(8):1998. doi: 10.3390/molecules23081998 (PMC6222902; doi:10.3390/molecules23081998)
Supplement: Supplementary file 1 [file molecules-23-01998-s001.pdf]

Supplementary Materials: **DPP-IV Inhibitory Potentials of Flavonol Glycosides Isolated from the Seeds of *Lens culinaris*: In Vitro and *Molecular Docking* Analyses**

Bo-Ram Kim [1], Hyo Young Kim [1], Inhee Choi [2], Jin-Baek Kim [1], Chang Hyun Jin [1], and Ah-Reum Han[1,*]

[1]Advanced Radiation Technology Institute, Korea Atomic Energy Research Institute, Jeongeup-si, Jeollabuk-do 56212, Republic of Korea; boram1606@kaeri.re.kr (B.-R.K.); khy5012@kaeri.re.kr (H.-Y.K.); jbkim74@kaeri.re.kr (J.-B.K.); chjin@kaeri.re.kr (C.H.J.); arhan@kaeri.re.kr (A.-R.H.)

[2]Institut Pasteur Korea, Seongnam-si, Gyeonggi-do 13488, Republic of Korea; inhee.choi@ip-korea.org (I.C.)

*Correspondence: arhan@kaeri.re.kr; Tel.: +82-63-570-3167

^{1}H-NMR Data of Compounds 1–3

Compound **1** (kaempferol-3-*O*-*β*-gulcopyranosyl-(1 → 2)-*β*-galactopyranosyl-7-*O*-*α*-rhamnopyranoside): Pale yellow solid. ESI-MS *m*/*z* 756.6 [M]$^{+}$. ^{1}H-NMR (500 MHz, CD_3OD, δ, ppm, *J*/Hz): 8.11 (2H, d, *J* = 9.0, H-2′ and H-6′), 6.90 (2H, d, *J* = 9.0, H-3′ and H-5′), 6.76 (1H, br s, H-8), 6.46 (1H, br s, H-6), 5.56 (1H, br s, H-1 of 7-*O*-Rha), 5.41 (1H, d, *J* = 7.5, H-1″ of 3-*O*-Gal), 4.75 (1H, d, *J* = 7.5 Hz, H-1 of 2″-*O*-Glc), 1.24 (3H, d, *J* = 5.0, H-6 of 7-*O*-Rha). ^{13}C-NMR data: see Table S1.

Compound **2** (kaempferol-3-*O*-*β*-gulcopyranosyl-(1→2)-[*α*-rhamnopyranosyl(1→6)]-*β*-galactopyranosyl-7-*O*-*α*-rhamnopyranoside): Pale yellow solid. ESI-MS *m*/*z* 902.8 [M]$^{+}$. ^{1}H-NMR (500 MHz, CD_3OD, δ, ppm, J/Hz): 8.06 (2H, d, *J* = 9.1, H-2′ and H-6′), 6.91 (2H, d, *J* = 9.1, H-3′ and H-5′), 6.75 (1H, br s, H-8), 6.48 (1H, br s, H-6), 5.56 (1H, br s, H-1 of 7-*O*-Rha), 5.36 (1H, d, *J* = 7.5, H-1″ of 3-*O*-Gal), 4.73 (1H, d, *J* = 7.5 Hz, H-1 of 2″-*O*-Glc), 4.47 (1H, br s, H-1 of 6″-*O*-Rha), 1.25 (3H, d, *J* = 5.5, H-6 of 7-*O*-Rha), 1.06 (3H, d, *J* = 5.5, H-6 of 6″-*O*-Rha). ^{13}C-NMR data: see Table S1.

Compound **3** (robinin; kaempferol-3-*O*-*α*-rhamnosyl(1→6)-*β*-galactopyranosyl-7-*O*-*α*-rhamnopyranoside): Pale yellow solid. ESI-MS *m*/*z* 740.6 [M]$^{+}$. ^{1}H-NMR (500 MHz, DMSO-d_6, δ, ppm, *J*/Hz): ^{1}H-NMR (500 MHz, CD_3OD, δ, ppm, J/Hz): 8.12 (2H, d, *J* = 8.5, H-2′ and H-6′), 6.87 (2H, d, *J* = 8.5, H-3′ and H-5′), 6.77 (1H, d, *J* = 1.5, H-8), 6.48 (1H, d, *J* = 1.5, H-6), 5.57 (1H, br s, H-1 of 7-*O*-Rha), 5.09 (1H, d, *J* = 7.5, H-1″ of 3-*O*-Gal), 4.51 (1H, br s, H-1 of 6″-*O*-Rha), 1.26 (3H, d, *J* = 6.0, H-6 of 7-*O*-Rha), 1.18 (3H, d, *J* = 6.0, H-6 of 6″-*O*-Rha). ^{13}C-NMR data: see Table S1.

Table S1: ^{13}C NMR data of compounds **1–3** (125 MHz, δ, ppm).

C atom	**1** (CD_3OD)	**2** (CD_3OD)	**3** (DMSO-d_6)
2	158.2	158.4	157.0
3	133.7	133.7	133.0
4	178.4	178.4	177.6
5	161.6	161.6	160.8
6	99.3	99.3	99.3
7	162.3	162.3	161.6
8	94.5	94.5	94.8
9	156.8	156.8	156.0
10	106.1	106.1	105.5
1′	121.3	121.3	120.6
2′	131.2	131.2	131.1
3′	115.0	115.0	115.1
4′	160.4	160.4	160.8
5′	115.0	115.0	115.1
6′	131.2	131.2	131.1
3-*O*-Gal			
1″	99.9	99.6	101.8
2″	81.2	81.2	71.1
3″	74.3	74.3	72.9
4″	69.9	69.9	68.2
5″	77.5	75.8	73.5
6″	61.5	66.8	65.0
2″-*O*-Gal			
1	103.4	103.4	
2	75.8	75.8	
3	76.6	76.6	
4	70.0	70.0	
5	76.9	76.9	
6	61.3	61.3	
6″-*O*-Rha			
1		100.8	100.0
2		70.8	70.4
3		71.0	70.6
4		72.5	71.9
5		68.4	67.9
6		16.5	17.9
7-*O*-Rha			
1	98.6	98.6	98.4
2	70.4	70.4	70.1
3	70.8	70.8	70.2
4	72.3	72.3	71.6
5	70.1	70.1	69.8
6	16.8	16.8	17.9
